# Evaluation of Membrane Integrity Monitoring Methods for Hollow Fiber Nanofiltration Membranes: Applicability in Gray Water Reclamation Systems

**DOI:** 10.1021/acsestwater.3c00307

**Published:** 2023-11-02

**Authors:** Samuel Benjamin Rutten, Bukola Lois Ojobe, Lucia Hernández Leal, Joris de Grooth, Hendrik D. W. Roesink, Jan Bartacek, Heike Schmitt

**Affiliations:** † Wetsus, European Centre of Excellence for Sustainable Water Technology, Oostergoweg 9, 8911 MA Leeuwarden, The Netherlands; ‡ Membrane Science and Technology, University of Twente, Drienerlolaan 5, 7522 NB Enschede, The Netherlands; § Department of Water Technology and Environmental Engineering, University of Chemistry and Technology Prague, Technicka 5, 166 28 Prague, Czech Republic; ∥ NXFiltration, Josink Esweg 44, 7545 PN Enschede, The Netherlands; ⊥ National Institute for Public Health and the Environment, Antonie van Leeuwenhoeklaan 9, 3721 MA Bilthoven, The Netherlands; # Department of Biotechnology, Delft University of Technology, Van der Maasweg 9, 2629 HZ Delft, The Netherlands; ¶ Institute for Risk Assessment Sciences, Faculty of Veterinary Medicine, Utrecht University, Heidelberglaan 8, 3584 CS Utrecht, The Netherlands

**Keywords:** hollow fiber, nanofiltration, source-separated sanitation, gray water reclamation, indirect membrane monitoring, antibiotic-resistant genes

## Abstract

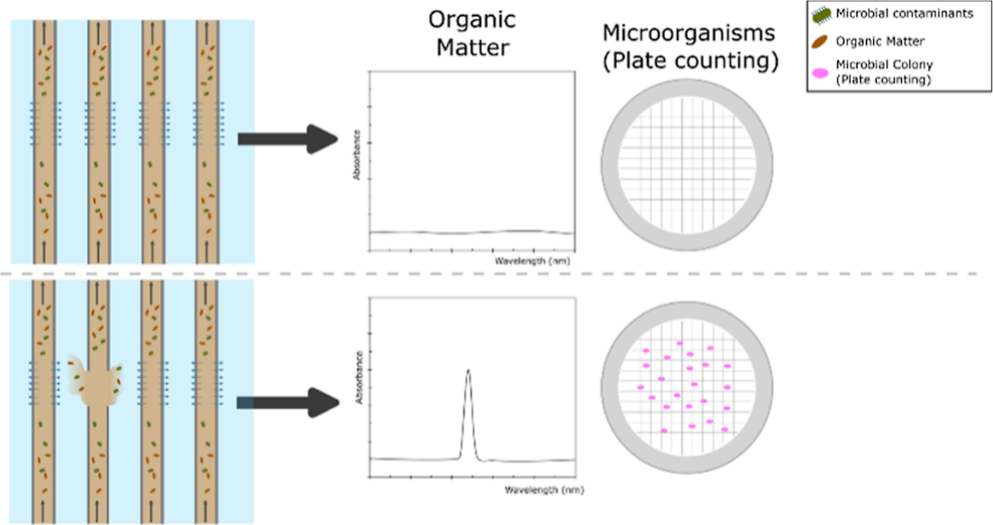

Source-separated gray water reclamation using nanofiltration as an advanced post-treatment option has received substantial interest in meeting the growing water demand. During reclamation, membrane integrity is crucial to ensure the water’s safety. This study evaluated several chemical and novel microbial indicators as indirect membrane integrity-monitoring methods for hollow fiber nanofiltration membranes in reclamation schemes. Under normal conditions, high retention of divalent ions and organic matter and near-complete removal of *Escherichia coli* (*E. coli*) were observed. Limited removal of the antibiotic gene (ARG) *tetO* was observed due to low feed concentrations and a higher detection limit (LOD). While 16S rRNA and ARG *sul1* were not limited by their LODs, lower removals were observed, most likely due to free-floating DNA passing through the membranes. A broken fiber in a pilot-scale module reduced organic matter and microorganism removal substantially, while flux and ion rejection remained similar. Predictions made using the observed results and a previously proposed model allowed for the evaluation of the selected methods in upscaled reclamation systems. Based on these results, it was concluded that microorganisms could be employed as indicators in indirect membrane integrity-monitoring methods in large-scale reclamation schemes, while UV_254nm_ absorbance (used in organic matter determination) could be a viable solution in pilot-scale systems.

## Abstract 2

The study provides insights into the application of indirect membrane monitoring methods in gray water reclamation processes. Experimental and modeled results lead to advice for the readers of ACS ES&T: Water to ensure effluent quality for safe water reuse processes.

## 1 Introduction

A viable strategy to meet the increasing domestic water demand is the reclamation of wastewater.^1,2^ When considering wastewater reclamation, source separation of domestically produced gray water could be of great interest since it omits toilet discharges. After biological treatment, gray water has previously been considered viable for nonpotable reuse applications.^3−5^ However, while most of the organic and nutrient loads are adequately removed during these processes, contaminants such as microorganisms, viruses, and micropollutants are not effectively treated and remain in the effluent.^5−9^ To address these contaminants before reuse, advanced post-treatment technologies are required.

Pressure-driven membrane filtration processes, such as nanofiltration (NF), have increasingly been considered a post-treatment option in wastewater reclamation.^2,9−12^ Studies have shown the applicability of these membranes by reporting near-complete removal of microorganisms and viruses under normal conditions as the apparent pore size of NF membranes of 0.5–1 nm is much smaller than the size of microbial contaminants, providing a suitable barrier.^13,14^

Membrane integrity is of the utmost importance when implementing membrane processes for wastewater reclamation. While membranes are effective barriers to potentially remove hazardous contaminants, a breakthrough can occur when the membrane system is damaged. These damages can occur due to several process factors. First, improper installation and maintenance can lead to damage to the modules.^2,9,15,16^ Second, aging by mechanical strain and chemical wear during normal operation could lead to losses in separation efficiency. Lastly, abrasion by particulates and sharp objects in the feed solution that are not removed in pretreatment could cause tears in the membrane surface, leading to direct contamination of the permeate by the feed solution. When these factors lead to excessive strain on a hollow fiber membrane, failure can occur, which mainly leads to flattening or cracking of a fiber in the module.^16^ Therefore, effective monitoring methods need to be implemented to reduce the potential of membrane failure that can affect the final produced water.

To ensure membrane integrity, regulations have been established with requirements to monitor membrane system integrity.^17^ These regulations stipulate criteria for all membrane filtration processes, which must be met to receive removal credits and ensure system safety. Removal credits are generally provided on a log scale (log removal values, i.e., LRVs) as these give a more pronounced difference at high removal rates. Furthermore, both continuous monitoring using indirect methods and verification using direct tests are set as minimum criteria to receive log removal credits.

During direct monitoring, the membrane surface and module casing are evaluated offline using methods that directly correlate with the integrity.^15^ These methods commonly consist of pressure-based tests such as pressure or vacuum decay tests, following standard practices such as the ASTM D3923-23 & D6908-06, where a direct correlation exists between observed trends in pressure decay and module integrity.^18−20^ Direct monitoring methods are highly sensitive to pinhole size breaches and glue defects but require significant downtimes and are labor-intensive. Therefore, these techniques are mostly considered economically unfavorable after the installation stage.^2,21^ Indirect membrane monitoring evaluates the removal of a specific contaminant to evaluate the membrane integrity. When significant changes in the removal of such contaminants are observed, integrity losses are assumed. Since no considerable downtimes are required during indirect membrane integrity monitoring, a slight preference for these methods exists. However, the efficiency of the technique highly depends on the surrogate’s concentration, baseline rejection, and analytical sensitivity.^18^

Several indirect monitoring methods have been proposed by previous research.^2,9,18,21−26^ Among physicochemical surrogate indicators, turbidity measurements and particle counting are regularly applied for indirect monitoring.^21^ While these methods are quick and relatively inexpensive, limited sensitivity due to low concentrations leaves them less applicable in high-pressure membrane processes.^22,23^ Conductivity and ion retention were also implemented to monitor membrane integrity. While able to be monitored online with LRVs up to log 3, sufficiently high concentrations are required to observe integrity losses.^18^ Organic matter monitoring using total organic carbon (TOC) analysis or UV_254nm_ spectroscopy has seen some implementation in online membrane integrity monitoring. Both TOC and UV_254_ absorbance have been shown to be sensitive enough to determine small leaks in nanofiltration membranes that treat brown lake water.^24^ Log removal values of ∼5.5 for both UV_254nm_ and TOC under normal operational conditions reduced to ∼log 2 due to leakage in both cases. Based on their observations, it was concluded that UV_254nm_ was most effective in monitoring NF membrane integrity for brown lake water treatment.^24^

In addition to chemical parameters, microbial contaminants have been used to monitor the membrane integrity. Plate counting of bacteria, such as *Escherichia coli* (*E. coli*), is considered the conventional approach^2,25^ While being an effective indicator, the application of plate counting is limited by the significant time delay between sampling and the result.^25^ Next to plate counting, flow cytometry (FCM) has been considered an alternative for water quality monitoring.^9,25^ Previous studies demonstrated the efficacy of FCM as a monitoring method and reported log removal values ranging between 2 and 4.5.^2,9,26,27^

More recently, quantification of microbial constituents indigenous to freshwater using quantitative polymerase chain reaction (qPCR) to monitor reverse osmosis membrane integrity has effectively been developed by Hornstra et al. (2019). Log removal values up to ∼8.5 were reported for intact modules, while log removal values dropped to ∼3.5 when a 1 mm hole was present in the membrane surface.^13^ Based on their results, it was concluded that the determination of indigenous microbial contaminants using qPCR could be of interest to evaluate log removal in water treatment systems.

The present study evaluated the applicability of multiple indirect membrane integrity monitoring methods in gray water reclamation systems. The suitability of indigenous integrity indicators, including novel antibiotic-resistant genes (ARGs) and previously proposed microbial and chemical substances, was investigated to determine their viability in gray water reclamation schemes. Both lab- and pilot-scale systems were used to assess the importance of equipment scale while developing and validating indirect monitoring methods. Additionally, the applicability of a model for integrity breached in hollow fiber membranes, proposed by Lidén et al. (2016), which uses hydraulic data to predict the contribution of a severed fiber to the permeate quality, was evaluated using acquired data. Lastly, the acquired results and the model were used to assess the potential effectiveness of the investigated indirect monitoring methods in up-scaled gray water reclamation systems.

## 2 Materials and Methods

### 2.1 Chemicals and Membranes

Before each experiment, biologically treated gray water effluent was collected from a source-separated treatment plant located in Sneek, The Netherlands. During the lab experiments, approximately 10 L of effluent was collected on the day of use to limit potential changes to the effluent during storage, while ∼180 L of biologically treated effluent was collected in a 200 L buffer tank to perform all pilot scale experiments. The average ionic composition and organic content, given as total organic carbon, of the effluent used throughout the current study is provided in Table 1.

**Table 1:** Ion Composition of the Biologically Treated Gray Water Effluent Located in Noorderhoek, Sneek

component	concentration mg·L^–^^1^
pH	7.6
Ca^2++^	50 ± 3
Mg^2+^	14 ± 1
Na^+^	149 ± 8
Cl^–^	93 ± 11
SO_4_^2–^	52 ± 8
PO_4_^2–^	14 ± 14
TOC (As C)	77 ± 43

All experiments were performed at least three times. The lab-scale experiments were executed to determine the baseline retention of the selected indicator contaminants and to verify the model proposed by Lidén et al. (2016).^24^ Experiments were performed by using a Mexplorer bench-scale unit acquired from NXFiltration (Enschede, The Netherlands), which was connected to a heat exchanger. An in-depth description of the setup is provided in our previous study.^28^ A lab-scale dNF40 module, kindly provided by NXFiltration, was used for all experiments. This membrane consists of a module with an approximate length of 30 cm. Each module contains ∼120 fibers (inner diameter = 0.7 mm), which are coated with a proprietary polyelectrolyte multilayer (PEM), leading to a membrane surface area of 0.065 m^2^ and a molecular weight cutoff (MWCO) of 400 Da. Before the experiments, the lab-scale module was flushed with ∼100 L of demi water at a cross-flow velocity (CFV) of ∼1.0 m s^–1^ to remove residual preservatives. The membrane was similarly cleaned between each experiment to remove any potential foulants from the module.

Pilot-scale experiments were performed to assess the effectiveness of the indicator contaminants in larger-scale systems. The experiments were performed using a Mexperience pilot system (NXFiltration) located at the source-separated wastewater treatment plant in Sneek, The Netherlands. A WMC110 dNF40 module with a membrane surface area of 14.5 m^2^, distributed over ∼4400, 150 cm long PEM-coated fibers was used during all experiments. Before the experiments, the pilot system was taken out of continuous operation and connected to a 200 L buffer tank to allow for complete recirculation of the concentrate and permeate. A schematic representation of the experimental setup is provided in the Supporting Information (S1). During the experiments, the biologically treated effluent was spiked with *E. coli* as a model organism. *E. coli* (6897 strain) was acquired from DSMZ GmbH (Braunschweig, Germany) and grown 1 day before each experiment to guarantee sufficient concentrations.

### 2.2 Experimental Protocols

#### 2.2.1 Lab-Scale Protocol

Before each experiment, *E. coli* was spiked to a final concentration of ∼10^7^ colony-forming units per milliliter (cfu mL^–1^). Following the addition of *E. coli*, samples were collected at 2, 4, and 6 bar transmembrane pressure (TMP), while the CFV was kept constant (0.4 m s^–1^). The membrane was broken by inserting a needle through one fiber on the inlet side. The break was confirmed by determining the flux at increasing TMPs and validating the results using the model described by Lidén et al. (2016).^24^ This model determines the contribution of a severed fiber to the permeate flow by using known operational settings such as the transmembrane pressure and hydraulic principles. A detailed description of the hydraulic model is provided in the Supporting Information (S2). Using the predicted flow through a breached membrane and the known feed and permeate concentration and flow through the membrane under normal conditions, the permeate concentration under breached conditions can be determined using eq 1.
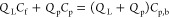
1where *Q*_L_ represents the water flow through the broken fiber, *C*_f_ is the feed concentration, *Q*_p_ and *C*_p_ are the permeate flow and concentration, respectively, and *C*_p,b_ is the mixed concentration of the permeate when a fiber is broken.

#### 2.2.2 Pilot Protocol

Prior to the experiment, a 200 L buffer tank was filled with ∼180 L of biologically treated gray water. Subsequently, the gray water was spiked with *E. coli*. to a concentration of ∼10^7^ cfu mL^–1^. To limit any potential concentration gradients, all experiments were performed in full recirculation (i.e., both the concentrate and permeate were returned to the buffer tank). Samples were collected at 2, 3, and 4 bar TMP, at a constant CFV of ∼0.4 m s^–1^. The full-scale module was breached by breaking one fiber with tweezers via the permeate port. Throughout the experiments, the flux was continuously monitored using *M*experience software and recorded during sampling.

### 2.3 Analytical Methods

Samples were analyzed for ions, organic matter, *E. coli*, and ARGs. Ion concentrations were determined using ion chromatography. The feed and permeate cationic and anionic composition were analyzed using a Metrohm Compact IC Flex 930 and Metrohm Compact IC 761 Ion chromatograph (Schiedam, The Netherlands). Ion concentrations were determined using a built-in conductivity probe. If required, samples were diluted to fit within the detection range provided in Supporting Information S3.

Organics were determined by using UV_254nm_ absorbance and total organic carbon (TOC) analysis. UV_254nm_ absorbance was determined by a Shimadzu UV-1800 (Shimadzu Benelux, ’s-Hertogenbosch, The Netherlands) using a quartz glass cuvette with a path length of 50 mm. Demineralized water was used as a blank, and sufficient time was provided to ensure steady absorbance. Samples were diluted when absorbance units exceeded ∼3. TOC concentrations were calculated by the difference between total carbon (TC) and inorganic carbon (IC), which was determined using a Shimadzu TOC-L Total Organic Carbon Analyzer (Shimadzu Benelux, ’s-Hertogenbosch, The Netherlands). Samples were diluted when the TC exceeded the detection range (>100 mg·L^–1^).

*E. coli* concentrations were determined using plate counting and qPCR. For plate counting, serially diluted samples were filtered with 0.45 μm filters (Merck, Darmstadt, Germany) and enumerated on Tryptone Bile X-glucuronide agar (TBX) according to the method of Blaak et al. (2021).^29^ For qPCR, samples were filtered using 0.22 μm PVDF Filters (Merck Millipore, Burlington, MA, USA). Sample volumes for DNA extraction varied between feed (20 to 50 mL) and permeate samples (20 to 500 mL) to adjust for the suspected *E. coli* concentration. DNA extraction was performed from filters by using a DNeasy PowerWater Kit (QIAGEN, Germany). The DNA extracts were analyzed for *ybbW* (*E. coli*), 16S rRNA, *sul1,* and *tetO*. Analysis was performed according to Pallares-Vega et al. (2021) using the primers and slight modifications provided in Supporting Information S4.^30^

## 3 Results and Discussion

### 3.1 Performance of Hollow Fiber NF under Normal Process Conditions

**Figure 1:**
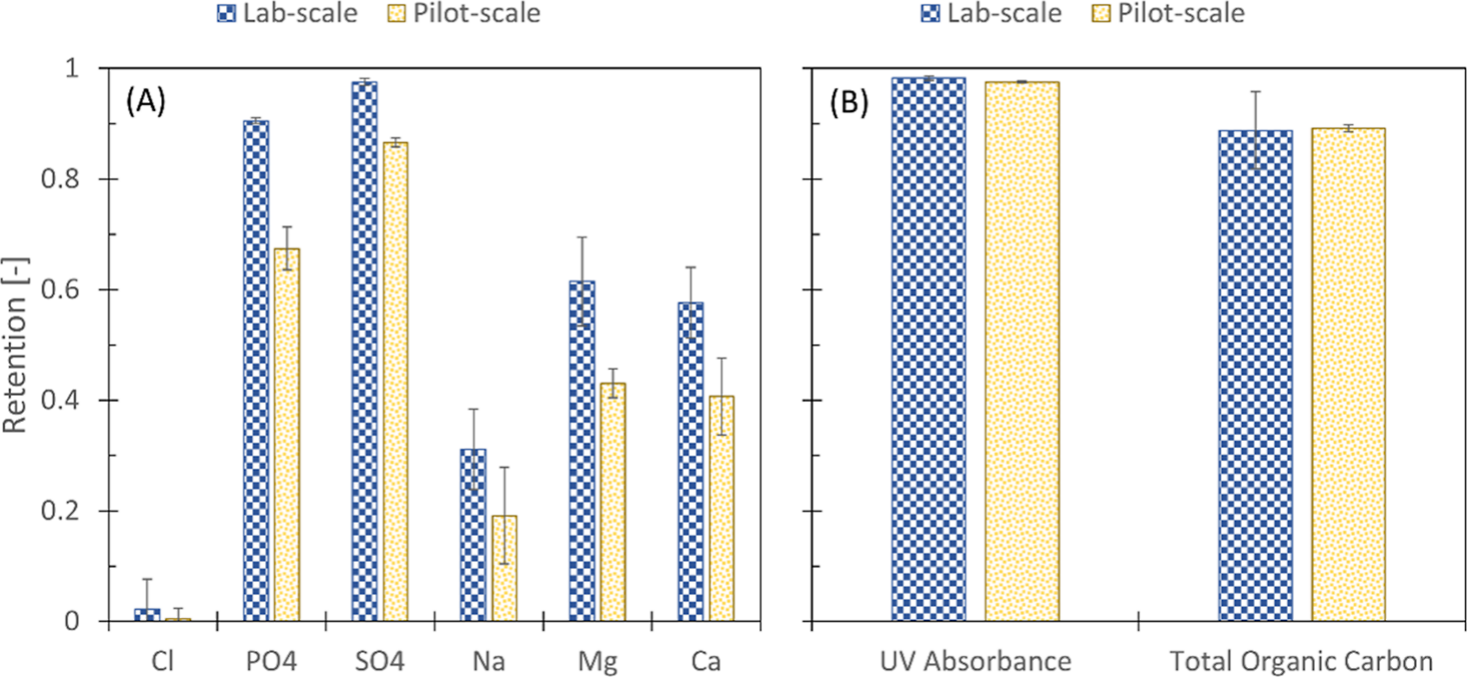
Retention of (A) ions and (B) organic matter from biologically treated gray water effluent by both lab-scale and pilot-scale dNF40 membranes.

Overall, a limited influence of transmembrane pressure on the retention of most contaminants was observed (Supporting Information S5). Therefore, the average retention of all studied solutes, irrespective of pressure, was determined (Figure 1).

The retention of most solutes was substantially lower at the pilot scale than the observed retention during the lab-scale experiments (Figure 1). This decrease in retention most likely occurred due to the increased length of the module and the operational settings during both experiments.^31^ The pilot-scale module was approximately five times longer than the lab-scale module. Due to this difference in dimensions, a disparity in the concentration gradients along the membrane surfaces could occur regardless of similar operational conditions. Even though the applied cross-flow velocity limits concentration polarization in the lab-scale modules, an increased concentration gradient along the membrane surface in the pilot module most likely occurred due to the increased length of the membrane fibers.^31^ Additionally, as higher water recoveries occurred at the pilot scale (∼50%) compared to the lab scale (∼3%), enhanced concentration build-up along the pilot module’s membrane surface was presumed to have occurred.^31^ This increased concentration at the feed side would lead to an increased concentration gradient across the membrane phase, enhancing solute transport and reducing the observed lower retentions.

In line with the expectations, a higher retention of divalent ions compared to monovalent ions was observed (Figure 1A). Due to the inherent negative charge of the dNF40 membranes, divalent anion retention, i.e., sulfate (Lab: 98 ± 1%; Pilot: 87 ± 1%) and phosphate (Lab: 91 ± 1%; Pilot: 67 ± 4%), exceeded divalent cation rejection (Lab: ∼ 60%; Pilot: ∼ 42% for both magnesium and calcium), while monovalent ion retention fluctuated between ∼0 and ∼30% regardless of scale. Based on these observations, it was presumed that divalent anions, more specifically sulfate, are most appropriate for further integrity monitoring tests due to their high retention and limited deviation.

While no substantial difference in organic matter retention between the lab-scale and pilot-scale experiments occurred, differences in organic matter retention based on the analytical method were observed (Figure 1B). Retention determined using UV_254nm_ (Lab: 98.2 ± 0.4%; Pilot: 97.5 ± 0.1%) substantially exceeded retention based on TOC analysis (Lab: 88.7 ± 7%; Pilot: 89.2 ± 0.7%). These differences in observed organic matter retention based on TOC and UV_254nm_ are most likely due to the more efficient removal of aromatic groups, which contribute more to absorbance at 254 nm.^24^ Considering that UV_254nm_ absorbance is a less laborious and expensive analytical technique and showed higher retentions than that of TOC analysis, it was expected that UV_254nm_ absorbance would be more appropriate as an indirect indicator method.

**Figure 2:**
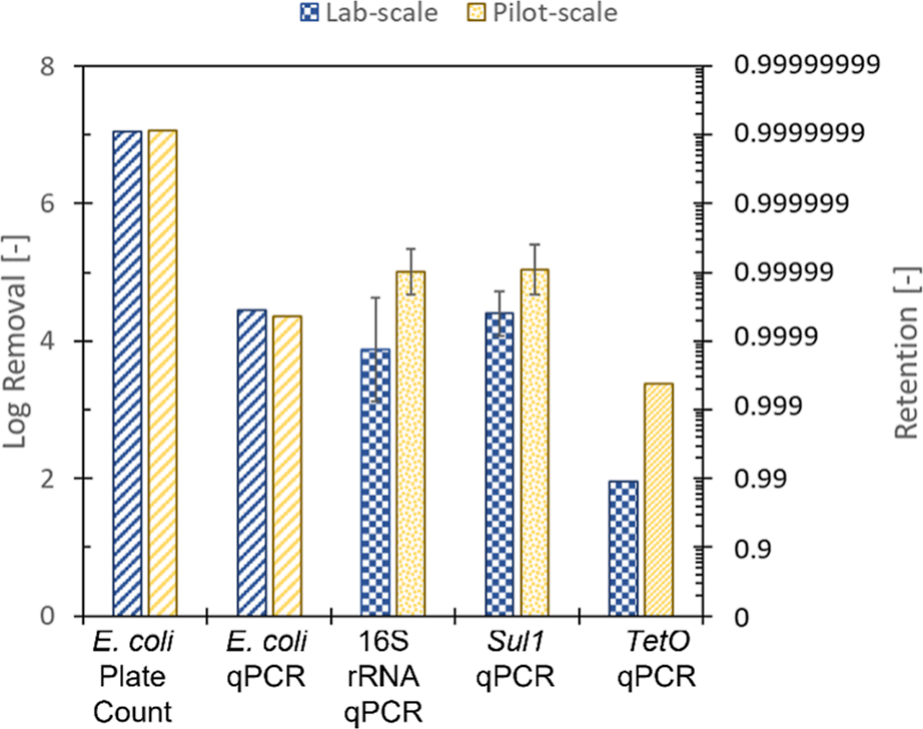
Log removal of *E. coli*, 16S rRNA, and ARGs (*sul1* and *tetO*) from spiked biologically treated gray water effluent. *E. coli* PC: removal was determined using plate counting. Striped bars represent a minimum log removal–actual log removal could not be determined as concentrations in the permeate were below the limit of detection (LOD).

Based on plate counting, near complete removal of *E. coli* was achieved since no colonies were detected in most of the undiluted permeate samples (Figure 2). Out of the nine permeate samples collected on the lab scale, only one sample provided a positive result with a log concentration of 0.18, which corresponded to a LRV of 6.88. On the pilot scale, all nine permeate samples did not contain any indication of *E. coli*. Due to this near complete absence of *E. coli* in the permeate samples, log removal values corresponded to the initial feed concentrations, as shown in Supporting Information S6. Near complete removal of *E. coli* was in line with the expectations as previous research reported removal of *E. coli* below detection limits using both ultrafiltration and nanofiltration.^2,32,33^ Krahnstöver et al. (2019) reported LRVs exceeding 5 for ultrafiltration membranes in wastewater reclamation schemes.^2^ Since nanofiltration membranes have a lower MWCO and pore size than ultrafiltration membranes, it is expected that well-operating high-pressure systems, such as nanofiltration systems, will result in similar or improved removal.^13^

Average log removals determined using qPCR were substantially lower than those determined using plate counting (Figure 2). For *E. coli* qPCR, most permeate samples targeting the *ybbW* gene were below the limit of detection (Supporting Information S6). While this was in line with the expectation based on plate counting, the relatively high limit of detection (LOD) of log 2.26 led to a limited determined log removal value of ∼4.4 on both lab and pilot scale modules. Since 16S rRNA is a nonspecific target for qPCR, higher concentrations in biological effluent were presumed to be present. While a higher initial 16S rRNA concentration was observed throughout most experiments, a substantial concentration of 16S rRNA, ranging from ∼3.2 on the lab scale and ∼2.8 on the pilot scale, was still present in the permeate. Previous work has shown the presence of significant portions of extracellular DNA which contributed to 16S rRNA qPCR results in wastewater effluent.^34^ 16S rRNA concentrations up to log 5 gene copies mL^–1^ from free-floating DNA were observed in conventional wastewater treatment effluents. While it is presumed that near complete retention of viable microorganisms can be achieved, passage of much smaller, elongated free-floating DNA could occur, leading to the observed lower log removals. Overall, an average 16S rRNA log removal of 3.9 ± 0.9 on the lab scale and 5.0 ± 0.3 on the pilot scale was achieved under normal operation.

In line with the expectation, both ARGs (i.e., *sul1* and *tetO*) were less prevalent in the effluent than both 16S rRNA and *ybbW*. Similar to previous observations, biologically treated gray water effluent contained higher concentrations of *sul1* (∼log 5.5 gene copies mL^–1^) than *tetO* (∼log 4 gene copies mL^–1^) (Supporting Information S6).^35^ While the dNF40 membrane effectively removed both ARGs, limited log removal values could be determined due to the relatively low feed concentration of each gene. Under normal operational conditions, most permeate samples contained low concentrations of *sul1* (Supporting Information S6), which led to observed removals of ∼4.4 on the lab scale and ∼5 on the pilot scale. *TetO* was removed below detection limits (log 0.84) in the permeate by the dNF40 membranes (Supporting Information S6), leading to LRVs of at least ∼ log 2 and ∼ log 3.4 on the lab and pilot scales, respectively. While these observations show the potential for gene-specific monitoring for membrane integrity, targeting more abundant genes needs to be considered and tested to determine its applicability. Considering the current results, it was expected that plate counting and *E. coli*-specific qPCR using the *ybbW* gene would be most effective for monitoring membrane integrity in larger-scale systems due to their higher measurable log removal values.

### 3.2 Performance of Indicator Contaminants in Breached NF Applications for Gray Water Reclamation

#### 3.2.1 Applicability of the Hydraulic Model for Integrity Breaches in Gray Water Reclamation Systems

**Figure 3:**
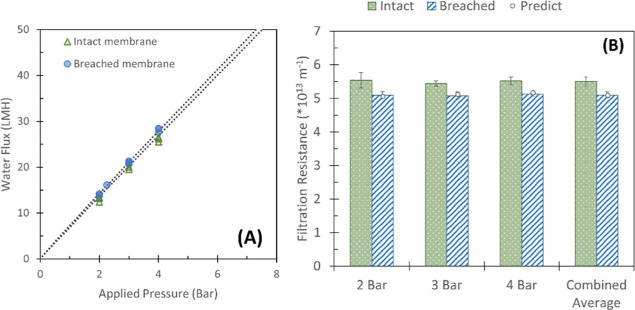
Change of flux due to a single fiber breach in a 4″ dNF40 module and its corresponding filtration resistance. (A) Water flux as a function of the applied pressure under normal operational conditions (green triangle) and under breached conditions (blue circle). The gray area between the dotted lines displays predicted flux change based on Lidén et al. (2016); (B) Average filtration resistance was observed under normal conditions (green) and breached conditions (blue). The gray circles represent the predicted loss in filtration resistance due to a single breach based on Lidén et al. (2016).

**Figure 4:**
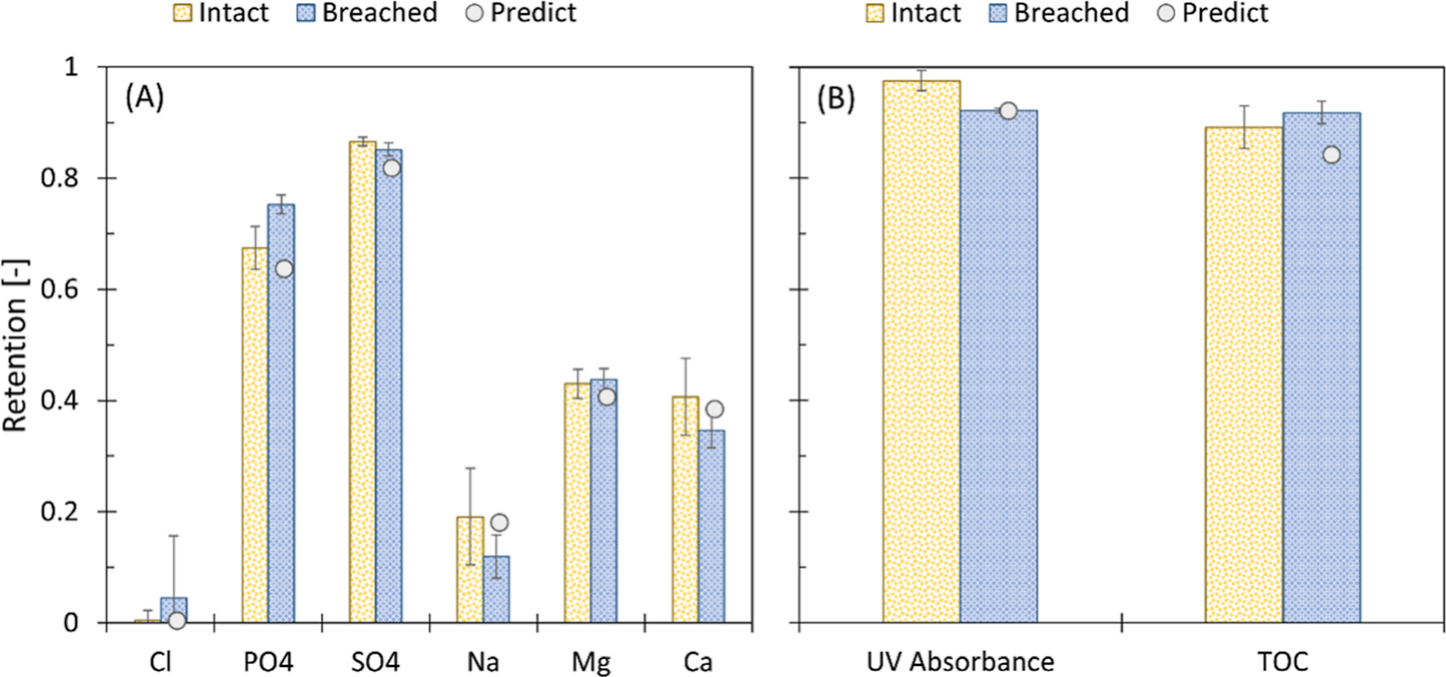
Retention of (A) ions and (B) organic matter by a 4 in. pilot-scale dNF40 membrane under normal and breached operation. Gray circles represent the change in retention predicted by the hydraulic model.

To assess the validity of the model proposed by Lidén et al. (2016), the change in flux due to a single breach in a lab-scale and pilot-scale module was determined and compared to the predicted increase in flux. Since a lab-scale module contains substantially fewer fibers than a pilot module (∼120 compared to ∼4400), breaks on the lab-scale are expected to lead to a more significant increase in permeate flow and subsequently lead to the near-complete passage of all targeted integrity monitoring substances. This substantial increase in permeate flow and near-complete passage of all contaminants was clearly observed and relatively well predicted (Supporting Information 7). Since the predictive model functioned relatively well at the lab scale, the model was further applied to the pilot module (Figures 3 and 4).

Since the pilot scale module contained ∼4400 fibers, one breach’s effect on both the flux and retention of the indicator contaminants was expected to be much less pronounced. While only a minor increase in the flux due to a breach was observed, a clear decrease in the filtration resistance was observed (Figure 3). Lidén et al. (2016) reported a higher sensitivity of filtration resistance toward integrity losses when compared to the water flux.^24^ The changes in flux and filtration resistance were relatively well predicted using the proposed model by Lidén et al. (2016), indicating its potential suitability to assess bigger systems using the acquired pilot results.

#### 3.2.2 Removal of Indicator Contaminants by a Compromised Pilot-Scale Module

No clear trend in the retention change for most chemical contaminants due to a single fiber breach was observed during the experiments (Figure 4). Insignificant changes in ion retention were observed during the pilot-scale experiments (Figure 4A). Most reductions remained within the standard deviation observed at normal conditions, limiting the use of ions as a membrane integrity indicator to set-ups smaller than the now applied pilot scale. The model mostly overpredicted the expected loss in retention, which was likely due to the variability observed during the normal conditions. While the loss in sulfate retention was relatively well predicted, its relatively large standard deviation under normal conditions limited the usability of the ion as an indicator contaminant. It must be noted that phosphate retention increased from 68 ± 4% during normal operation to 75 ± 2% when the membrane was breached. This increase in observed retention most likely occurred due to an increased analytical accuracy as feed concentrations of phosphate increased 10-fold between the experiments under normal conditions and breached conditions. Since the permeate concentrations under normal conditions were close to the detection limits of 0.1 mg·L^–1^, the observed retentions were limited by this LOD. Due to the 10-fold increase in feed phosphate concentrations during the experiments with the breached module, a subsequent increase in permeate concentrations was observed, regardless of the breach. This increase in permeate concentration eliminated the potentially limited observed retention due to the LOD, and it is presumed to have led to the observed increase in PO_4_ retention (Figure 3A).

In terms of organic matter removal, a clear decrease in UV_254nm_ absorbance, from 97.5 ± 0.1% to 92.2 ± 0.5%, was observed (Figure 4B). While there was a relatively small decrease of ∼5%, high stability in the observed absorption change led to limited deviation in the results. Furthermore, the predicted retention (92.1%) agreed well with the observed loss in retention, indicating its potential use in bigger-scale systems. Due to a relatively large deviation in organic matter retention determined using TOC analysis, no significant change in retention could be observed. These results were consistent with the observations of Lidén et al. (2016), where a higher sensitivity of UV_254nm_ absorbance for membrane integrity monitoring compared to TOC-analysis was observed.^24^ Based on the observations for all studied chemical contaminants, it was concluded that UV_254nm_ absorbance would be a more viable membrane integrity monitoring method in gray water reclamation schemes due to its lower variance, potential direct measurement, ease of implementation, and scalability.

**Figure 5:**
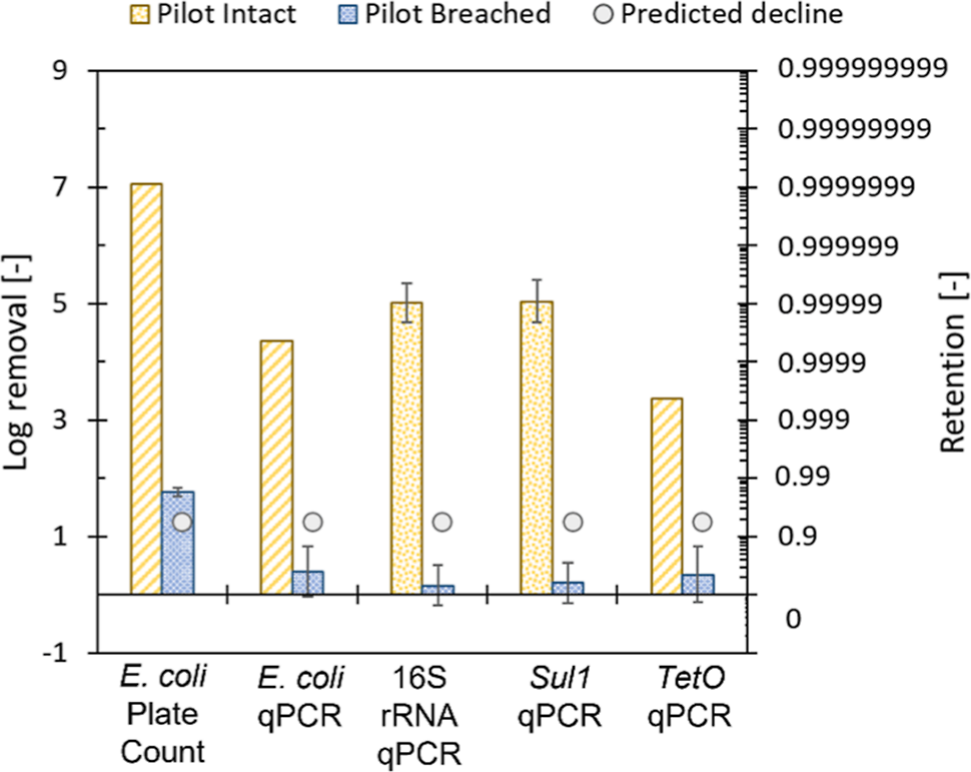
LRVs of microbial indicators by a 4″ pilot scale dNF40 membrane under normal and breached conditions. The striped bar graphs represent the log removal values based on the LOD, while the gray circles represent the predicted loss in log removal values.

A single breach in the pilot-scale module clearly led to significant losses in log removal values observed for all microbial indicators (Figure 5). Using plate counting, a reduction in log removal values from ∼7.6 to 1.7 ± 0.1 was observed, clearly displaying the viability of plate counting as an integrity monitoring method in large-scale set-ups. The modeled result slightly overpredicted the contribution of one single fiber breach toward the loss in LRVs by ∼log 0.5. The loss in log removal values of all targeted genes using qPCR exceeded both the observed loss for plate counting and the predicted value. The LRV of the *E. coli*-specific gene, *ybbW* reduced from ∼log 4.4 to log 0.4 ± 0.4 due to a singular fiber breach, while 16S rRNA reduced from ∼5 to log 0.16 ± 0.35. The removal of the novel ARGs showed a similar trend, where *sul1* removal reduced from ∼5 to log 0.20 ± 0.35 and *tetO* removal reduced from ∼3.4 to 0.35 ± 0.48 (Figure 5). The loss in LRV for all microbial indicators targeted by qPCR was underpredicted by ∼log 0.85 (*ybbW*) to log 1.75 (*tetO*). This underprediction could be caused by several effects, such as matrix effects caused by sample composition, artifacts in the qPCR method, or misalignment of the model parameters. Based on the current results, no conclusive reason for the misalignment of the model for the microbial indicators could be provided, and future studies to enhance the quality of the predictions are recommended. Considering the observed changes in LRV due to a single fiber failure for all targeted microbial indicators, it was presumed that plate counting (LRV change log ∼5.9), qPCR targeting 16S rRNA (LRV change log ∼4.8), and *sul1* (LRV change log ∼4.8) would currently be the most appropriate as indicators in pilot-scale gray water treatment systems.

#### 3.2.3 Implications for Integrity Monitoring in Multimodule Installations

**Figure 6:**
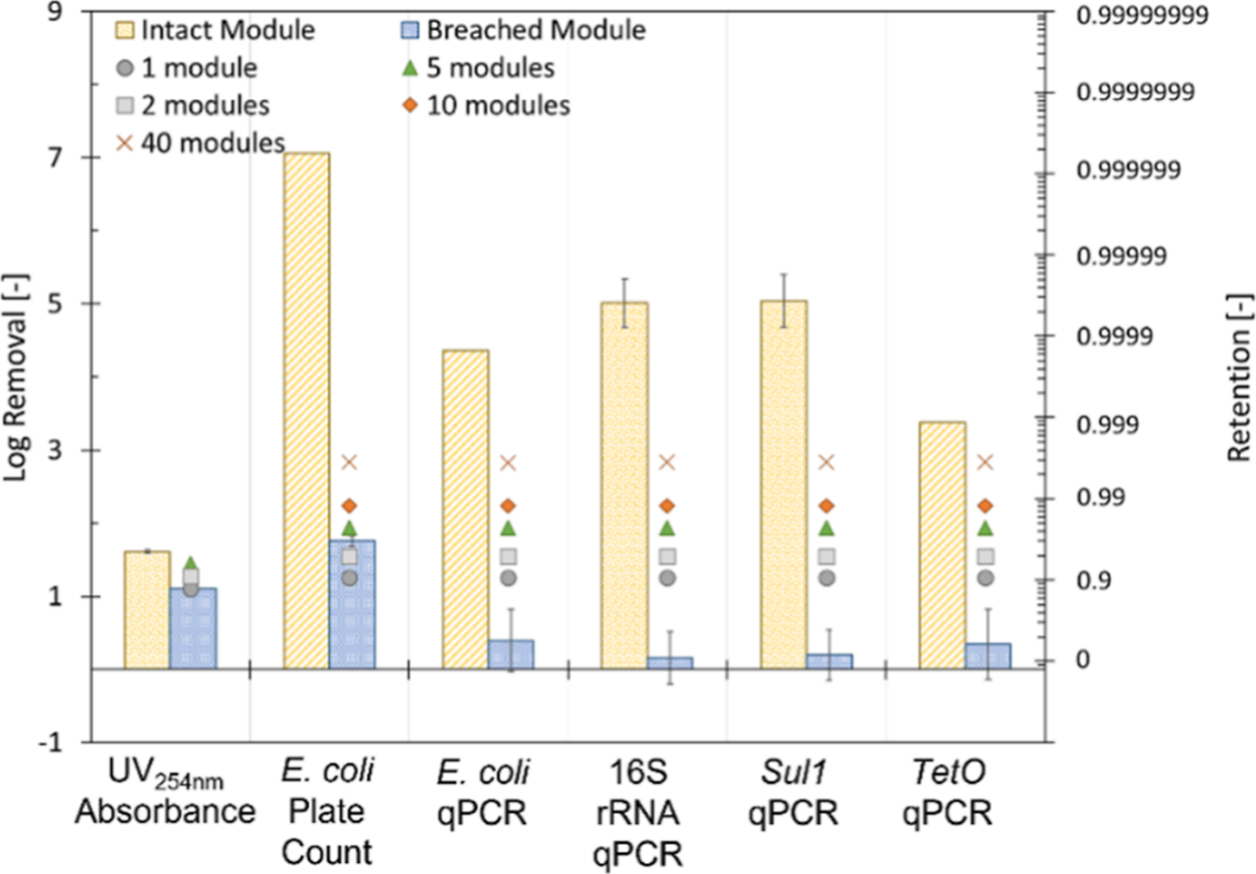
Log removal of organic matter determined by UV_254nm_ absorbance and the microbial indicators (*E. coli* by plate counting and qPCR, 16S rRNA, *sul1,* and *tetO*) by an intact and a breached (single fiber) 4″ dNF40 module. The markers represent the predicted log removal values based on the number of extra 4″ inch modules using the hydraulic model and mass balance. The secondary *y*-axis presents retention values, which align with the log removal values.

Since log removal values, in essence, represent the retention of constituents on a log scale, it was decided to express all indicators of interest (UV_254nm_ absorbance and all microbial indicators) in log removal values (Figure 6). Furthermore, even though the model underestimated the loss in log removal values, it was still attempted to assess the change in LRVs in multimodule systems. To evaluate the model results for the better-predicted indicators (i.e., UV_254nm_ absorbance and *E. coli* plate counting), in a conservative approach, a minimum required change of 0.1 LRVs, including standard deviation, was chosen as the limit to observe and determine a breach.

As previously shown in Figure 4, a single breach was only clearly observed using UV_254nm_ absorbance as a physical-chemical indicator. Therefore, UV absorbance was the only chemical indicator considered during the theoretical assessment of multimodule systems. Using the observed log removal of UV and the predicted change in log removal in multimodule systems, the range of applicability of UV absorbance as an indirect membrane integrity monitoring method was estimated (Figure 6). Based on the predictions, UV_254nm_ absorbance could still be a viable membrane integrity monitoring method up to a maximum of five 4-in. modules (*A*_mem_ = ∼72.5 m^2^). This would allow the application of UV absorbance in pilot-scale and small-scale industrial setups that do not exceed five modules. However, large-scale gray water reclamation installations will most likely consist of systems with more than five modules, limiting the applicability of UV absorbance as an integrity monitoring solution. Furthermore, since online UV absorbance sensors are relatively expensive, the method’s viability from an economic point of view is rather quickly lost. For *E. coli* determined by plate counting, the model predicted the observed log removals sufficiently. It was, therefore, presumed that predictions of multimodule systems could provide some insight. Based on the modeled changes in log removal values in multiscale systems, a single broken fiber in 40 dNF40 modules (∼580 m^2^) would most likely still be detectable using plate counting (Figure 6).

Considering the experimental observations, plate counting and qPCR are viable methods to assess the membrane integrity indirectly. While the reduction in log removal by plate counting could be predicted reasonably well with the model, substantial deviations in the modeled results and the observed reduction in log removal by qPCR were observed. Therefore, the modeled results could only be considered qualitatively for qPCR when assessing multimodule systems. Based on the qualitatively modeled results, higher observed LODs, and the limited presence of some of the targeted genes (i.e., *tetO*) in biologically treated effluent, qPCR is expected to be more limited than plate counting currently. Since permeate contamination caused by a broken fiber will decrease with an increasing number of modules, LOD will become a limiting factor at a certain point. Since plate counting has the most extensive range between initial concentration and LOD (Supporting Information S8), it should currently still be preferred when assessing membrane integrity.

While plate counting is currently the most reliable solution to determine a loss in membrane integrity due to its lower LOD, the time required to obtain the result substantially lowers its overall practicality. Therefore, further improvements of qPCR, such as a reduction in the detection limits and the development of inline measurement equipment, could enhance the applicability as a membrane integrity monitoring solution in future gray water reclamation systems.

## 4 Conclusions

Membrane integrity is of utmost importance when it is applied in wastewater reclamation schemes. In the current study, monitoring of potential fiber breaches in hollow fiber nanofiltration membranes was investigated in gray water reclamation schemes using a multitude of indicator contaminants.

Under normal operating conditions, effective retention of divalent ions and organic matter was observed, while near complete removal of *E. coli* was achieved by both lab-scale and pilot-scale dNF40 membranes. 16S rRNA and *sul1* were observed in the permeate, possibly due to free-floating DNA passing through the membrane.

A break in a single fiber highlighted the importance of the equipment scale when developing and validating indirect membrane integrity monitoring methods. All targeted contaminants fully passed through the lab-scale module, while no discernible differences in the retention of ions and organic matter (by TOC analysis) at the pilot scale were observed.

A single broken fiber in a pilot module was effectively detected by UV_254nm_ spectroscopy, plate counting, and qPCR, highlighting their potential viability as supplementary indirect membrane integrity monitoring methods alongside conventional pressure-based decay tests.

The modeled results suggest that UV_254nm_ absorbance could be an effective membrane integrity monitoring solution for pilot to small-scale industrial applications that do not exceed membrane surface areas over ∼72 m^2^. Microbial indicators are most likely able to assess substantially larger gray water reclamation systems with membrane installations that exceed membrane surface areas of 500 m^2^. While qPCR, using indigenous microbial indicators such as ARGs, is a promising technology that could eventually replace conventional microbial-based membrane integrity monitoring solutions (i.e., plate counting), limitations, such as relatively high detection limits, lead to plate counting presently being more successful in detecting fiber failures in large-scale reclamation schemes.

## Supporting Information Available

Schematic overview of the pilot-scale setup, description of the predictive model, details of the chemical and microbial analyses, additional results including pressure-dependent retention, concentration range using plate counting and qPCR, flux and retention changes for the lab-scale membranes under breached conditions, changes in permeate concentrations based on the model, and equations to determine retention, log removal values, and water recovery (PDF)
