# Supplementary material for: Evaluation of Membrane Integrity Monitoring Methods for Hollow Fiber Nanofiltration Membranes: Applicability in Gray Water Reclamation Systems
Source: ACS ES T Water. 2023 Nov 2;3(12):3884–92. doi: 10.1021/acsestwater.3c00307 (PMC10714397; doi:10.1021/acsestwater.3c00307)
Supplement: Supplementary file 1 — ew3c00307_si_001.pdf [file ew3c00307_si_001.pdf]

Evaluation of membrane integrity monitoring methods for hollow fiber nanofiltration membranes:

Applicability in greywater reclamation systems.

Samuel Benjamin Rutten[a,b*], Bukola Lois Ojobe[a,c], Lucia Hernández Leal[a*], Joris de Grooth[b,d], Hendrik D.W. Roesink[b,d], Jan Bartacek[c], Heike Schmitt[a,e,f,g]

[a] Wetsus, European Centre of Excellence for Sustainable Water Technology, Oostergoweg 9, 8911 MA, Leeuwarden, The Netherlands

[b] Membrane Science and Technology, University of Twente, Drienerlolaan 5, 7522 NB, Enschede, The Netherlands

[c] Department of Water Technology and Environmental Engineering, University of Chemistry and Technology Prague, Technicka 5, 166 28, Prague, Czech Republic

[d] NXFiltration, Josink Esweg 44, 7545 PN, Enschede, The Netherlands

[e] National Institute for Public Health and the Environment, Antonie van Leeuwenhoeklaan 9, 3721 MA, Bilthoven, The Netherlands

[f] Department of Biotechnology, Delft University of Technology, Van der Maasweg 9, 2629 HZ, Delft, The Netherlands

[g] Institute for Risk Assessment Sciences, Faculty of Veterinary Medicine, Utrecht University, Heidelberglaan 8, 3584 CS, Utrecht, The Netherlands

* Corresponding Authors:	Lucia Hernández Leal -	email: lucia.hernandez@wetsus.nl;
	Samuel Benjamin Rutten -	email: s.b.rutten@utwente.nl

S1. Schematic overview of the pilot scale set-up

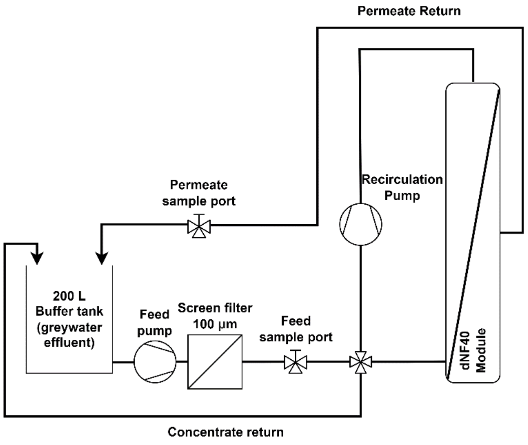

Figure S1. Schematic overview of the pilot-scale mexperience system.

S2. Predictive model

A simple model predicting the change in TOC concentration due to a compromised fiber was proposed previously (Lidén et al., 2016). Within this study's scope, this model's applicability in predicting retention changes for all indirect monitoring compounds was evaluated. While an extensive description of the hydraulic model is provided in Lidén et al. (2016), a summarised version is provided in the following paragraphs.

In the case of an intact membrane, flow through the membrane, Q_p ($L.h^{-1}$), can easily be determined when the water permeability, P ($L.m^{-2}.h^{-1}.Bar^{-1}$), is known.

$$Q_p = PA_{mem}(\Delta TMP - \Delta\Pi) \tag{1}$$

Where A_{mem} represents the known membrane surface area (m^2), and ΔTMP and $\Delta\Pi$ represent the pressure drop over the membrane and the osmotic pressure in Bar. Since membrane area and the water permeability are constants, the flow through the membrane is, in this case, only dependent on the nominal pressure applied to the membrane. During this process, the retention (R) of solutes can be determined using equation 2, where C_p and C_f are the permeate and feed concentrations, respectively.

$$R = 1 - \frac{C_p}{C_f} \tag{2}$$

When a fiber is breached, the flow to the permeate side and the permeate concentration will change. Normally, the flow through a membrane depends on the membrane's resistance and

the pressure difference over the membrane surface. During a breach in a fiber, the membrane's resistance will disappear, and an open flow from the feed to the permeate, dependent on the pressure drop between the feed and permeate side, will occur. Since this pressure drop simulates the flow through a capillary channel, the flow through the broken fiber can be calculated using a modified version of the Hagen-Poiseuille flow through a laminar pipe formula (Equation 3).

$$Q_B = A_{fiber}\sqrt{\frac{2\Delta TMP}{\rho_{a+\frac{fs}{d}}}} \tag{3}$$

In this, Q_B is the flow through the broken fiber from one side, A_{fiber} is the cross-sectional area of one fiber, ρ the water density, α a kinetic energy correction coefficient, f the friction factor, s the point of breakage downstream from the inlet, and d the inner diameter of the broken fiber. α was determined based on the flow regime, in which laminar flow (Reynold <2000), α was determined to be 2, and in turbulent flow (Reynolds >4000), α was 1. In the transition zone, α was changed linearly from 2 to 1. The friction factor (f) was similarly dependent on the flow regime and determined using either equations 3, 4 or 5.

$$f = \frac{64}{Re} \qquad Re < 2000 \tag{4}$$

$$f = 0.0115\ln(Re) - 0.0557 \qquad 2000 < Re < 4000 \tag{5}$$

$$\frac{1}{\sqrt{f}} = 0.8684\ln\left(Re\sqrt{f}\right) - 0.8 \qquad Re > 4000 \tag{6}$$

Since both sides of the broken fiber will develop a pressure drop, flows coming from both the feed and the concentrate side need to be considered when determining the effect of a break on the flow. Therefore, Q_B will need to be calculated for both the feed and concentrate side of the module and added to a total flow from a leak, Q_L. An example of the dependency of the flow on the position of the break and the transmembrane pressure is provided in Figure S2.

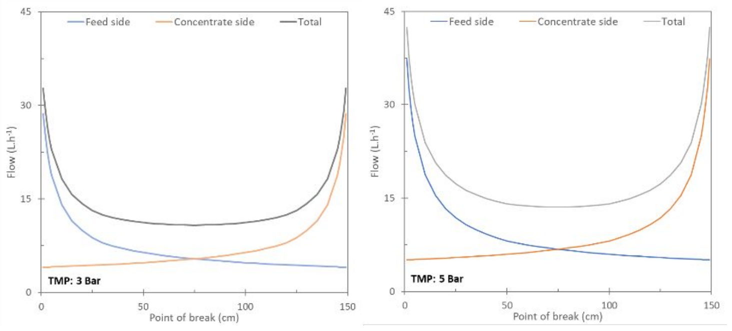

Figure S2. Flow through a broken fiber at different transmembrane pressures. Total fiber length: 150 cm at cross-flow velocity: 0.4 m.s^{-1}.

In regular operation, the flow through the membrane and the concentrations of the feed and permeate are known. When fiber breakage occurs, the permeate concentration will change following a simple mass balance.

$$Q_L C_f + Q_p C_p = (Q_L + Q_p) C_{p,b} \tag{7}$$

Where $C_{p,b}$ is the mixed concentration of the permeate with the flow of a broken fiber. The number of broken fibers can be determined since Q_L for one breach can be approximated, and C_m can be determined by analysis.

S3. Chemical analysis – Ion chromatography

Cations were determined in isocratic mode using a Metrohm Metrosep C-4 column with a Methrohm Metrosep RP 2 Guard pre-column and a 3 mM nitric acid aqueous mobile phase. Anions were separated by a Metrohm Metrosep A Supp 5 with a Methrohm Metrosep A Supp 4/5 pre-column. Anions were separated in isocratic mode using a mobile phase containing 1% acetone, 3.2 mM sodium carbonate, and 1 mM sodium bicarbonate. Both cation and anion concentrations were determined using a built-in conductivity detector. If required, samples were diluted to fit within the detection range (Table S1).

Table S1. Detection Limits Ion chromatography

Compound	Detection range ($mg.L^{-1}$)
**Sodium**	0.1 – 20
**Calcium**	0.1 – 20
**Magnesium**	0.1 – 20
**Chloride**	0.1 – 20
**Sulphate**	0.1 – 20
**Phosphate**	0.1 – 20

S4. Microbial analysis – Real Time PCR Analysis

All qPCR analysis were conducted on a Applied Biosystems 7500 Real-Time PCR Systems (Applied Biosystems, USA)) with 20 µL total reaction volume and HOT FirePol EvaGreen qPCR supermix (Solis Biodyne) and 1 ul (E. coli) or 2 µL DNA template (16S rRNA, *sul1*, and *tetO).* Primers are specified in Table S2. For the detection of E. coli by PCR, a fragment of the putative Allantoin transporter for the ybbW gene was targeted. Double stranded gBlocks Gene Fragments (integrated DNA Technologies) were used as control templates, with template genes separated by repetitive T sequences.. The concentration of the standards was confirmed using fluorometry.

Table S2. Oligonucleotide primer sequences used in the current study

Target	Primer	Primer Sequence 5'- 3'	Primer length (bp)	Sequence Length (bp)	Source
E. coli.[a]	401F<br>611R	F:TGATTGGCAAAATCTGGCCG<br>R:ATGGCGATTTGGGCGATTTC	20<br>20	280	Walker et al. (2017)
**16s rRNA**[b]	q_1114F<br>q_1275R	F:CGGCAACGAGCGCAACCC<br>R:CCATTGTAGCACGTGTGTAGCC	18<br>22	145	Denman & McSweeney, 2006
Sul1[b]	q_sulI_FW<br>q_sulI_RV	F:CGCACCGGAAACATCGCTGCAC<br>R:TGAAGTTCCGCCGCAAGGCTCG	22<br>22	162	Pei et al. 2006
TetO[b]	tetO_Fw<br>tetO_Rv	F: ACGGARAGTTTATTGTATACC<br>R: TGGCGTATCTATAATGTTGAC	21<br>21	142	Zhang et al., 2017

The qPCR thermal cycling conditions for *E. coli* detection were as follows: 5 min at 94 °C(initial polymerase activation and denaturation), followed by 40 cycles of one min 94 °C, a minute at 55^{0}C, and a minute at 72^{0}C, afterward, an extension step of 5 min at 72 °C (Walker et al.,2019). For 16S rRNA gene detection, the qPCR thermal cycling conditions were as follows 15 min at 95 °C (initial polymerase activation and denaturation), followed by 40 cycles of one min 95 °C, a minute at 55 °C, 30 seconds at 72 °C and final polymerization at 72 °C for 8 mins. For the detection of *tetO*, the qPCR thermal cycling conditions were as follows 15 min at 95 °C (initial polymerase activation and denaturation), followed by 40 cycles of one min 95 °C, a minute at 60 °C, 30 seconds at 72 °C and final polymerization at 72 °C for 8 mins. To ensure the quality of the qPCR process, the PCR cycle threshold (CT) was set to 28 and defined as the limit of detection. For a gene to be said to be detected in a sample, both technical duplicates had to be amplified.

S5. Pressure dependent retention under normal conditions

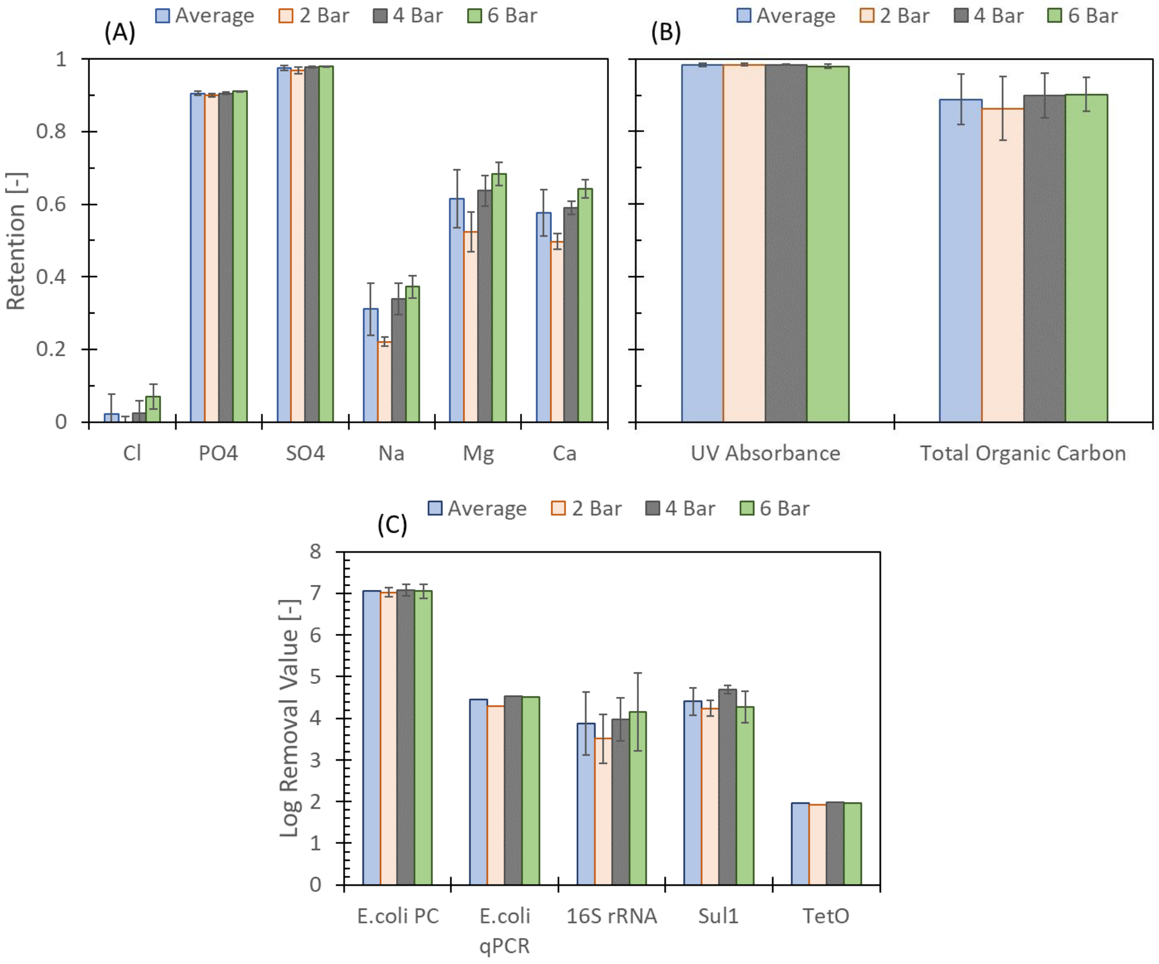

Figure S3. Pressure-dependent retention of the selected indicators by a lab-scale dNF40 module under normal conditions.

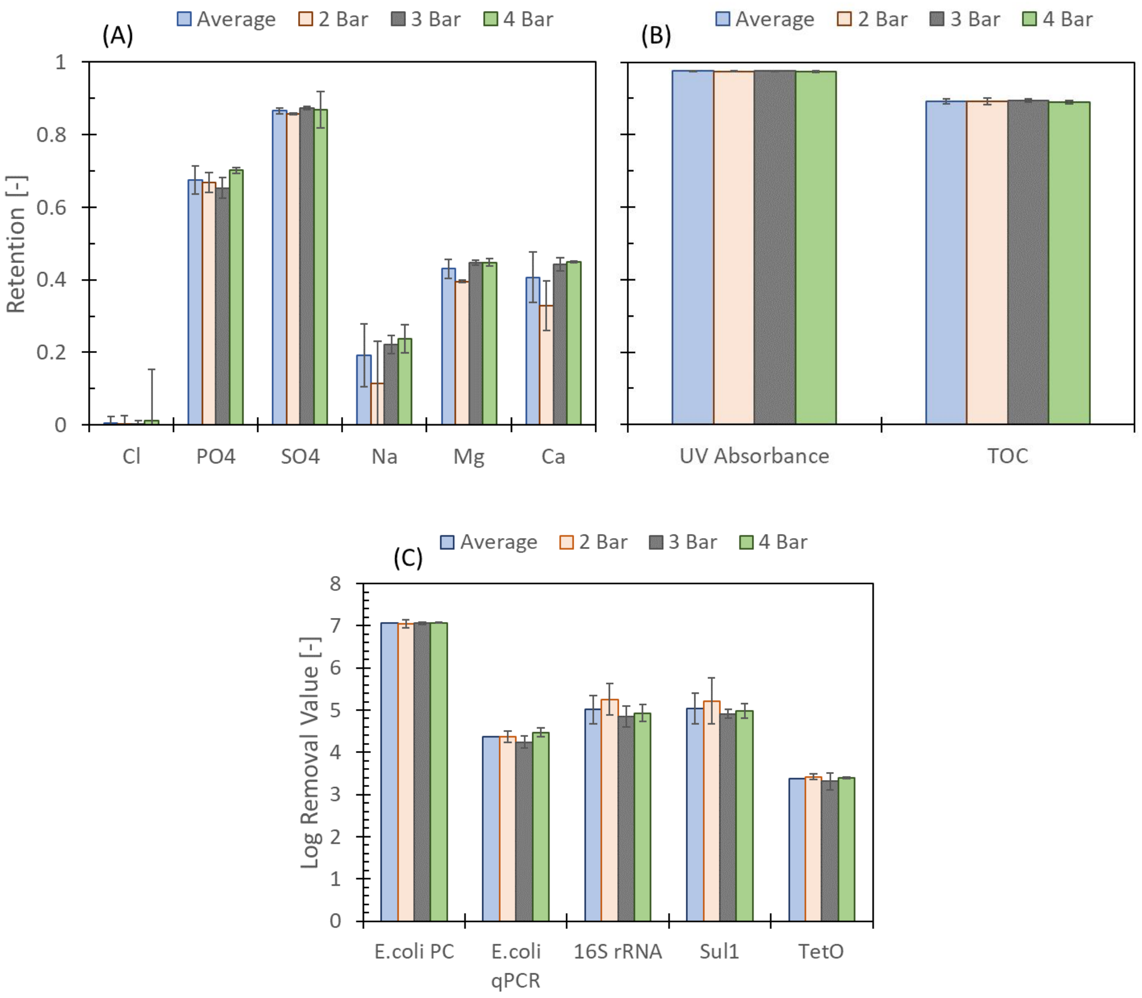

Figure S4. Pressure-dependent retention of the selected indicators by a pilot-scale dNF40 module under normal conditions.

S6. Concentration range plate counting and qPCR under normal conditions

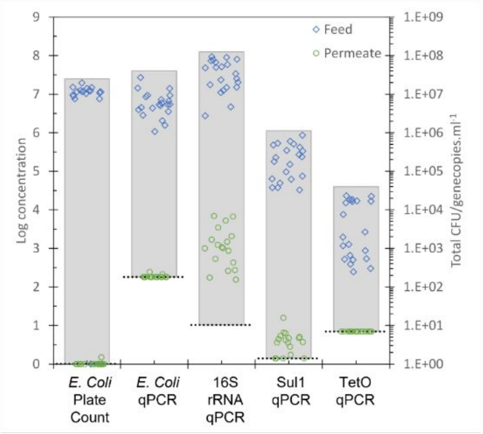

Figure S5. Observed concentration range of *E. coli* (using Plate counting & qPCR) 16S rRNA, *sul1* and *tetO* throughout all experiments

S7. Flux and retention changes observed when a lab-scale membrane was damaged

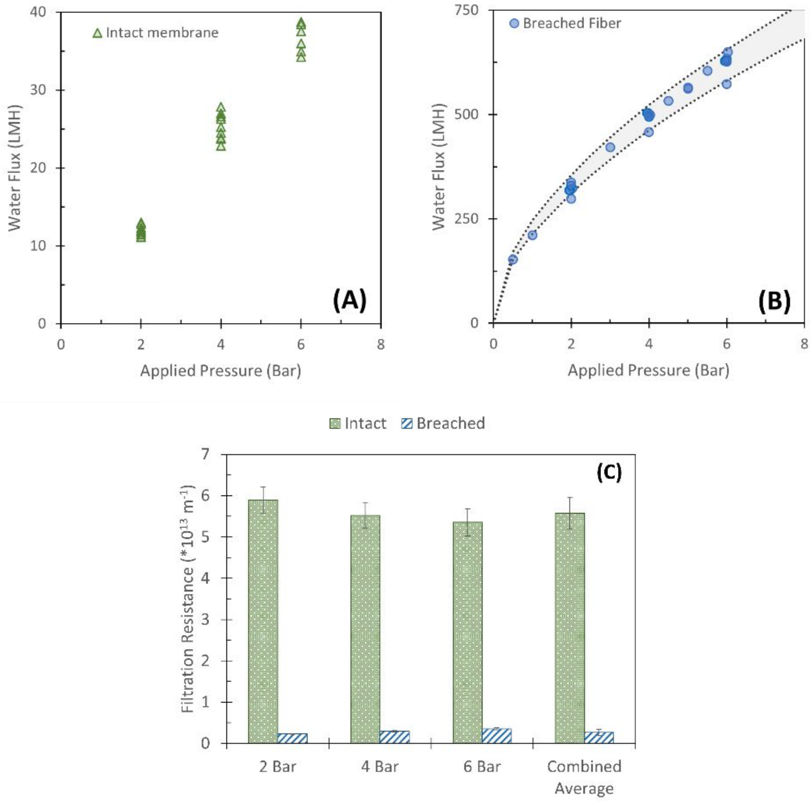

Figure S6. Water flux through an intact and breached lab-scale dNF40 module and its corresponding filtration resistance. (A): Water flux as a function of applied pressure during normal operational conditions; (B) Observed water flux as a function of applied pressure with one singular broken fiber. The marked grey area between the dotted lines illustrates the expected range of increase in flux due to one breached fiber; (C) the average observed filtration resistance as a function of applied pressure during normal operation (green) and under breached conditions (blue).

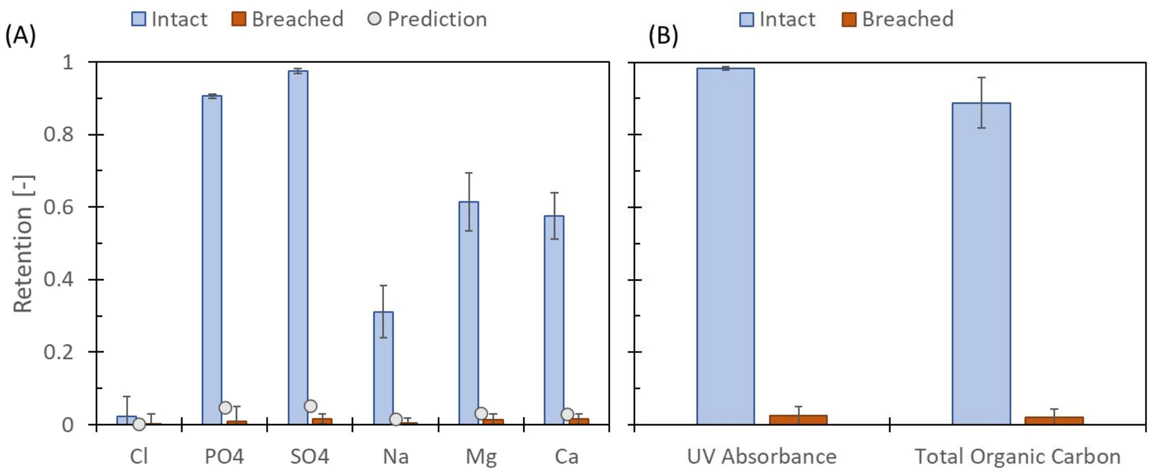

Figure S7. Retention of indicator contaminants by lab-scale dNF40 membranes (A_{mem}=0.065 m^2) with and without a breach. Error bars represent the standard deviation.

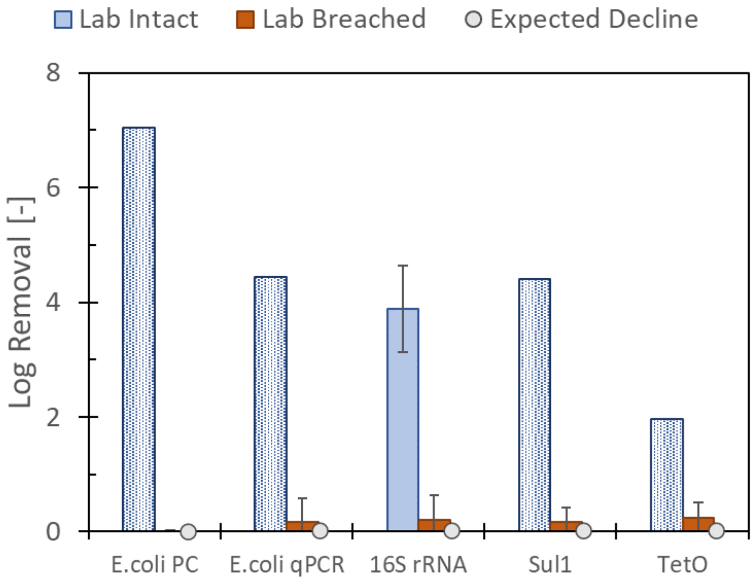

Figure S8. LRVs the microbial inidcators determined by plate counting and qPCR using a lab-scale dNF40 membrane (A_{mem}=0.065 m^2) with and without a breach. Error bars represent the standard deviation.

S8. Change in permeate concentration of microbial indicators based on model

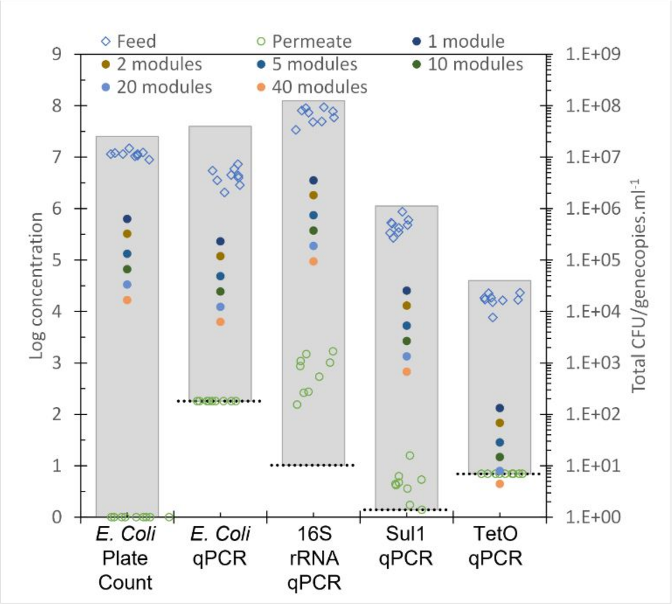

Figure S9. Concentration of all microbial indicators at the pilot scale, and the predicted concentration of each indicator due to a breach in larger scale systems.

S9. Equations used to determine retention, log removal values and water recovery.

Throughout the manuscript, both the retention and log removal values of the indicators substances are provided. Both parameters represent the ratio of a certain contaminant concentration in the feed (c_{feed}) and permeate ($c_{permeate}$), and can be calculated using equations 8 and 9.

$$Retention = 1 - \frac{c_{permeate}}{C_{feed}} \quad (8)$$

$$Log\ removal\ value = Log10\left(\frac{C_{feed}}{C_{permeate}}\right) \quad (9)$$

Furthermore, the water recovery on the lab-scale and pilot scale was determined to assess the effect of concentration build-up. The water recovery is based on the fraction of produced permeate ($Q_{permeate}$) and the supplied feed flow (Q_{feed}) and is determined by equation 10.

$$Water\ Recovery\ (\%) = \frac{Q_{permeate}}{Q_{feed}} * 100 \quad (10)$$

S10. References

Denman, S. E., & McSweeney, C. S. (2006). Development of a real-time PCR assay for monitoring anaerobic fungal and cellulolytic bacterial populations within the rumen. *FEMS Microbiology Ecology*, *58*(3), 572–582. https://doi.org/10.1111/J.1574-6941.2006.00190.X

Pei, R., Kim, S. C., Carlson, K. H., & Pruden, A. (2006). Effect of River Landscape on the sediment concentrations of antibiotics and corresponding antibiotic resistance genes (ARG). *Water Research*, *40*(12), 2427–2435. https://doi.org/10.1016/J.WATRES.2006.04.017

Walker, D. I., McQuillan, J., Taiwo, M., Parks, R., Stenton, C. A., Morgan, H., Mowlem, M. C., & Lees, D. N. (2017). A highly specific Escherichia coli qPCR and its comparison with existing methods for environmental waters. *Water Research*, *126*, 101–110. https://doi.org/10.1016/J.WATRES.2017.08.032

Zhang, C., Xu, L., Wang, X., Zhuang, K., Liu, Q., Chong-Miao Zhang, C., & Wang, X. C. (2017). Effects of ultraviolet disinfection on antibiotic-resistant Escherichia coli from wastewater: inactivation, antibiotic resistance profiles and antibiotic resistance genes. *Journal of Applied Microbiology*, *123*(1), 295–306. https://doi.org/10.1111/JAM.13480

Lidén, A., Lavonen, E., Persson, K. M., & Larson, M. (2016). Integrity breaches in a hollow fiber nanofilter – Effects on natural organic matter and virus-like particle removal. Water Research, 105, 231–240. https://doi.org/10.1016/j.watres.2016.08.056
